# Comparison of the Neuroprotective Effects of Aspirin, Atorvastatin, Captopril and Metformin in Diabetes Mellitus

**DOI:** 10.3390/biom9040118

**Published:** 2019-03-27

**Authors:** Maryam Paseban, Reza Mohebbati, Saeed Niazmand, Thozhukat Sathyapalan, Amirhossein Sahebkar

**Affiliations:** 1 Department of Physiology, School of Medicine, Mashhad University of Medical Sciences, Mashhad 9177948564, Iran; pasebanm921@mums.ac.ir (M.P.); mohebbatir931@mums.ac.ir (R.M.); 2 Student Research Committee, Faculty of Medicine, Mashhad University of Medical Sciences, Mashhad 9177948564, Iran; 3 Neurogenic Inflammation Research Center, Mashhad University of Medical Sciences, Mashhad 9177948564, Iran; amir_saheb2000@yahoo.com; 4 Department of Academic Diabetes, Endocrinology and Metabolism, Hull York Medical School, University of Hull, Hull HU3 2RW, UK; Thozhukat.Sathyapalan@hyms.ac.uk; 5 Biotechnology Research Center, Pharmaceutical Technology Institute, Mashhad University of Medical Sciences, Mashhad 9177948564, Iran; 6 School of Pharmacy, Mashhad University of Medical Sciences, Mashhad 9177948564, Iran

**Keywords:** diabetes, oxidative stress, metformin, captopril, atorvastatin, aspirin, neuroprotective

## Abstract

Objective: The aim of this study was to investigate the effect of combined intake of a high dose of aspirin, atorvastatin, captopril and metformin on oxidative stress in the brain cortex and hippocampus of streptozotocin (STZ)-induced diabetic rats. Material and methods: Rats were randomly divided into the following 11 groups: control and diabetic (D), as well as 9 groups that were treated with metformin (M, 300 mg/kg) or aspirin (ASA, 120 mg/kg) alone or in different combinations with captopril (C, 50 mg/kg) and/or atorvastatin (AT, 40 mg/kg) as follows: (D + M), (D + ASA), (D + M + ASA), (D + M + C), (D + M + AT), (D + M + C + ASA), (D + M + C + AT), (D + M + AT + ASA) and (D + M + C + AT + ASA). The rats in treatment groups received drugs by gavage daily for six weeks. Serum lipid profile and levels of oxidative markers in the brain cortex and hippocampus tissues were evaluated. Results: The levels of malondialdehyde in the brain cortex and hippocampus in all the treated groups decreased significantly (*p* < 0.05). There was a significant increase in the total thiol concentration as well as catalase activity in treated rats in (M + AT), (M + C + ASA), (M + C + AT), (M + AT + ASA) and (M + C + AT + ASA) groups in cortex and hippocampus in comparison with the diabetic rats (*p* < 0.05). Also, the superoxide dismutase activity in all treated rats with medications was significantly increased compared to the diabetic rats (*p* < 0.05–0.01). Conclusion: Our findings showed that the combined use of high-dose aspirin, metformin, captopril and atorvastatin potentiated their antioxidant effects on the brain, and hence could potentially improve cognitive function with their neuroprotective effects on hippocampus.

## 1. Introduction

Diabetes mellitus (DM) is a metabolic disease with multi-organ involvement including kidney, heart and even the brain [1,2]. There is growing evidence that oxidative stress is a possible mechanism in the development of diabetes-related complications. It has been shown that glucose autoxidation along with activated polymorphonuclear cells in diabetes results in oxidative stress via hydroxyl and superoxide radicals generation [3].

Metformin, atorvastatin, aspirin and captopril are the frequently used medications in people with diabetes. The role of these medications on oxidant–antioxidant balance in diabetes is crucial. In this study, we investigated the role of these medications on the redox system.

Metformin is the first line anti-hyperglycemic agent used in the management of patients with type 2 diabetes [4]. Metformin reduces intracellular reactive oxygen species (ROS) levels by potentiating the activity of the antioxidant enzymes [5].

Dyslipidemia is commonly associated with DM [6], and statins are the most commonly used medications to treat this. Statins (e.g., atorvastatin) are lipid-lowering drugs [7], which reduce the risk of cardiovascular complications. Also, statins have multiple pleiotropic effects [8,9,10,11,12] including antioxidant activity via inhibition of NAD(P)H oxidase and free radical scavenging activity [13,14,15].

Aspirin (ASA) is another drug that is commonly used for secondary prevention of cardiovascular events in patients with diabetes [16]. Aspirin acts as an anti-oxidant by reducing the generation of multiple free radicals such as superoxide and by preventing reduction of antioxidant enzymes activity such as catalase and superoxide dismutase [17].

In addition, captopril, an Angiotensin II (Ang II) converting enzyme (ACE) inhibitor, is commonly used for secondary prevention of cardiovascular events in patients with diabetes. It can selectively lower the Ang II, endothelin and oxidative stress, which may have a potential role in its blood pressure-lowering effect [18].

There is some evidence to show that there is an increased risk of development of diabetes associated with statin use, and there is a renewed interest to find novel therapeutic approaches for managing hyperlipidemia. In addition, there is some evidence to show that high dose of aspirin may potentially have a role in lipid metabolism. Also a high dose of aspirin, unlike statin, can potentially ameliorate insulin resistance and improve glucose tolerance in patients with type 2 diabetes [19]. In view of this, there are ongoing studies on the use of high dose of aspirin in patients with diabetes. On the other hand, the effects of combinations of drugs on human health have recently become important since many patients take multiple medications simultaneously [20]. Since there has been no study on the effect of combined administration of atorvastatin, captopril, metformin and a high dose of aspirin on oxidative stress in the brain tissue, we studied this effect in a streptozotocin (STZ)-induced diabetic model.

## 2. Materials and Methods

### 2.1. Chemicals and Drugs

All drugs were purchased from Sigma (Darmstadt, Germany). Aspirin, CAS No. 50-78-2; atorvastatin, CAS No. 134523-00-5; metformin, CAS No. 657-24-9; captopril, CAS No. 62571-86-2; and streptozotocine, CAS No. 55325-01-4.

### 2.2. Streptozotocin-Induced Diabetes

For diabetes induction, a single dose of streptozotocin (60 mg/kg) was dissolved in distilled water and injected intraperitoneally (i.p.). Three days after the STZ injection, we evaluated fasting glucose levels in blood samples provided from the tail vein using a glucometer to certify the induction of diabetes. Rats with blood glucose level of ≥250 mg/dL were classified as diabetic [21].

### 2.3. Animals

Healthy adult male Wistar rats (250–280 g, 10 weeks old) were housed under standard conditions with 12-h light–dark cycle at a temperature 22 ± 2 °C with free access to food and water. All tests were carried out under license from the Animal Experimentation Ethics Committee of the Mashhad University of Medical Sciences (approval code: IR.MUMS.FM.REC. 1395.200, approval date: 20 July 2016).

### 2.4. Experimental Groups

Rats were randomly divided into the following 11 groups (8 animals in each group): control and diabetic (D), as well as 9 groups that were treated with metformin (M, 300 mg/kg) or aspirin (ASA, 120 mg/kg) alone or in different combinations with captopril (C, 50 mg/kg) and/or atorvastatin (AT, 40 mg/kg) as follows: (D + M), (D + ASA), (D + M + ASA), (D + M + C), (D + M + AT), (D + M + C + ASA), (D + M + C + AT), (D + M + AT + ASA) and (D + M + C + AT + ASA). The rats in treatment groups received daily drugs by gavage for 6 weeks. Serum lipid profile and levels of oxidative stress markers in the brain cortex and the hippocampus tissues were evaluated. All drugs were dissolved in saline.

### 2.5. Measurement of Total Thiol

The total thiol was determined in line with the methods of Sedlak and Lindsay [22]. The gastric and liver tissues of control and treated rats were removed and homogenised in ice-cold water and then were centrifuged. The supernatant (50 μL) was added to 1 mL Tris-EDTA (ethylenediaminetetraacetic acid) buffer (pH 8.6) and the absorbance was read at 412 nm against Tris-EDTA buffer alone (A1). Then, 20 μL of 5,5’-dithiobis-(2-nitrobenzoic acid) (DTNB; 10 mM in methanol) was mixed with the supernatant and the absorbance was read again (A2). The absorbance of DTNB reagent was also read as blank (B). The total thiol was expressed as mmol/g tissue.

### 2.6. Measurement of Malondialdehyde

Malondialdehyde (MDA) was measured using thiobarbituric acid (TBA) as described by Mihara et al. [23]. One millilitre of the supernatant of the homogenised gastric and liver tissues was added to 2 mL of a complex solution containing TBA, trichloroacetic acid (TCA) and hydrochloric acid (HCl). This was then boiled in a water bath for 40 min. After reaching room temperature, the solution was centrifuged at 1000 *g* for 10 min. The absorbance was read at 535 nm. The MDA levels were expressed as µmol/g tissue.

### 2.7. Determination of Superoxide Dismutase (SOD) Activity

SOD activity was assayed by Madesh and Balasubramanian method. A colorimetric assay involving superoxide production by pyrogallol auto-oxidation and the inhibition of superoxide-dependent reduction of the tetrazolium dye, MTT (3-(4, 5-dimethylthiazol-2-yl) 2, 5-diphenyltetrazolium bromide) to its Formazan by SOD was evaluated at 570 nm. The quantity of enzyme causing 50% inhibition in the MTT decline rate was determined as one unit of SOD activity [24].

### 2.8. Measurement of Catalase Activity

Catalase activity was determined by applying the procedure described by Zini et al. by reducing the concentration of H_2_O_2_ when incubated with the test samples. The quantity of tissue capable to decline the amounts of H_2_O_2_ existing in solution by 50% was determined as one unit of catalase-like activity [25].

### 2.9. Lipid Profile Assessment

Serum cholesterol, triglycerides, low-density lipoprotein cholesterol (LDL-C) and high-density lipoprotein cholesterol (HDL-C) were measured on day 0 and 45 using Pars Azmoon kits (Karaj, Iran).

### 2.10. Data Analysis

The results were expressed as mean ± standard error of the mean (SEM). Statistical analysis was performed by one-way ANOVA followed by the Tukey test. *p* < 0.05 was considered significant.

## 3. Results

**Figure 1:**
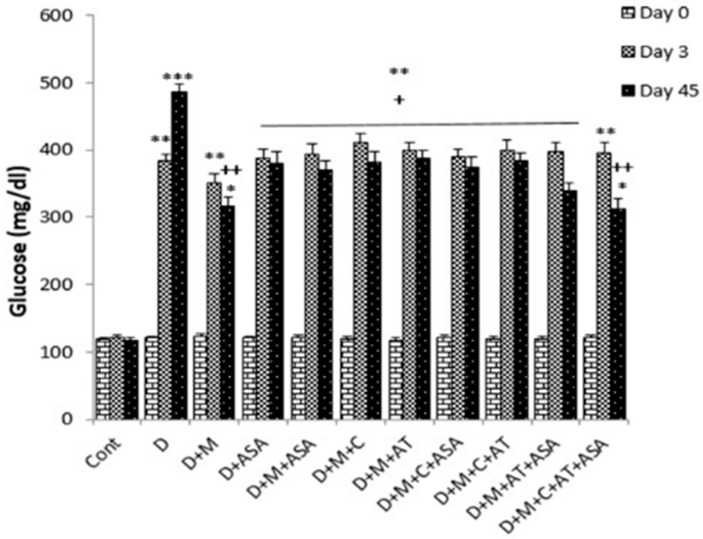
Glucose levels on days 0, 3 and 45 of the experimental period. Data are shown as mean ± standard error of the mean (SEM). * *p* < 0.05, ** *p* < 0.01 and *** *p* < 0.001 compared to control. + *p* < 0.05 and ++ *p* < 0.01 compared to diabetic group. D, diabetic; M, metformin; ASA, aspirin; AT, atrovastatin; and C, captopril.

Serum glucose concentration in the diabetic group and all groups treated with metformin and aspirin alone or different combinations of metformin, aspirin, atorvastatin and captopril were significantly higher than the control group. On day 45, this amount in all drug-treated groups was significantly lower than diabetic group (*p* < 0.05 to 0.01) (Figure 1).

**Figure 2:**
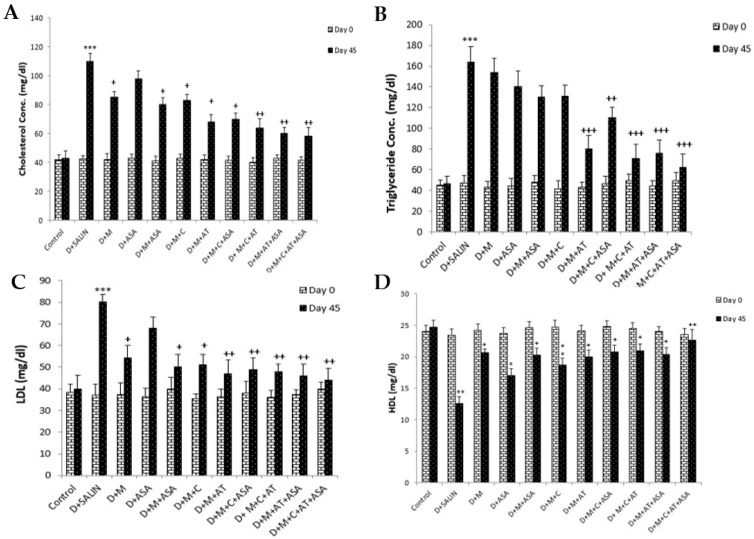
Serum levels of cholesterol (**A**), triglyceride (**B**), LDL-cholesterol (**C**) and HDL-cholesterol (**D**). Data are shown as mean ± SEM. * *p* < 0.05, ** *p* < 0.01 and *** *p* < 0.001 compared to control group, and + *p* < 0.05, ++ *p* < 0.01 and +++ *p* < 0.001 compared to diabetic group. D, diabetic; M, metformin; ASA, aspirin; AT, atrovastatin and C: captopril.

On day 45, serum total cholesterol concentration in the diabetic group was significantly higher compared to the control group (*p* < 0.001). All drug-treated groups, except the aspirin-only-treated group, showed a significant reduction in serum cholesterol concentration compared to the diabetic group (*p* < 0.05 to *p* < 0.01). Groups which received combinations of three or four drugs showed a significant reduction in serum cholesterol compared to groups received one or two drugs (*p* < 0.05 to *p* < 0.01). Among the three groups that received combinations of two drugs, a significant decline in serum cholesterol concentration was observed in (D + M + ASA) and (D + M + AT) groups, compared to the (D + ASA) group (Figure 2A).

There was a significant increase in serum triglyceride concentration in the diabetic group compared to the control group on day 45 (*p* < 0.001). Serum triglyceride concentration in groups which received combinations of three or four drugs was significantly lower than the diabetic group (*p* < 0.01 to 0.001). Among the three groups treated with combinations of two drugs, only the (D + M + AT) group showed a significant reduction in serum triglyceride concentration compared to the diabetic group (*p* < 0.001). Also, triglyceride concentration in groups that received combinations of three or four drugs was significantly reduced as compared to groups treated with one drug or combinations of two drugs (Figure 2B).

On day 45, serum LDL concentration was significantly higher in the diabetic group compared to the control group (*p* < 0.001). All drug-treated groups, except the ASA group, showed significant decreases in serum LDL concentration compared to the diabetic group (*p* < 0.05 to 0.01). LDL concentration in all groups treated with different combinations of drugs were significantly lower compared to ASA group (*p* < 0.05) (Figure 2C).

In the diabetic group, serum HDL concentration on day 45 was significantly lower than those of the control group (*p* < 0.01). All drug-treated groups, except the ASA group, showed a significant increase in serum HDL concentration compared to the diabetic group (*p* < 0.05 to 0.01). HDL concentration indicated significant increases in the groups which received a combination of four drugs compared to the ASA group (*p* < 0.05) (Figure 2D).

**Figure 3:**
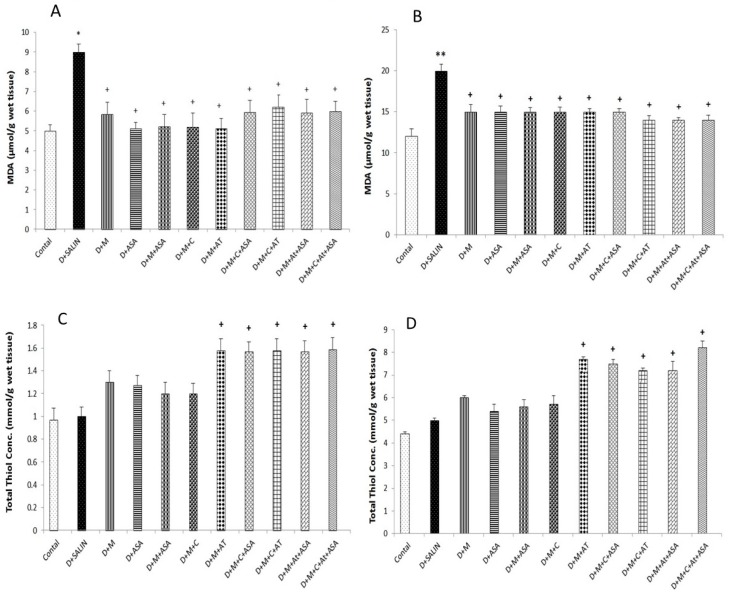
Malondialdehyde (MDA) and total thiol concentrations in the cortex (**A**,**C**) and hippocampus (**B**,**D**) tissues. Data are shown as mean ± SEM. * *p* < 0.05 and ** *p* < 0.01 compared to control. + *p* < 0.05 compared to non-treated diabetic group. D, diabetic; M, metformin; ASA; aspirin; AT, atorvastatin; and C, captopril.

Malondialdehyde level was increased in the cortex (*p* < 0.05) and hippocampus (*p* < 0.01) tissues in the diabetic group compared to the control group. Administration of captopril, aspirin, atorvastatin, metformin and their combinations reduced MDA level significantly compared to the diabetic group (*p* < 0.05) (Figure 3A,B).

The total thiol concentration in cortex and hippocampus in rats treated with (M + AT), (M + C + ASA), (M + C + AT), (M + AT + ASA) and (M + C + AT + ASA) significantly increased in comparison with the diabetic rats (*p* < 0.05) (Figure 3C,D).

**Figure 4:**
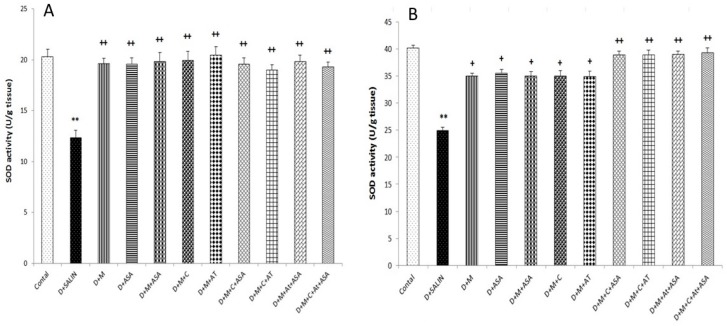
Superoxide dismutase (SOD) activity in the brain cortex (**A**) and hippocampus (**B**) tissues. Data are shown as mean ± SEM. ** *p* < 0.01 compared to control group, + *p* < 0.05 and ++ *p* < 0.01 compared to diabetic group. D, diabetic; M, metformin; ASA, aspirin; AT, atorvastatin; and C, captopril.

Also, the SOD activity in diabetic rats significantly decreased compared to the control group (*p* < 0.01). Superoxide dismutase activity boosted significantly in all groups treated with captopril, aspirin, atorvastatin, metformin and their combinations compared to the diabetic rats (*p* < 0.05 to *p* < 0.01) (Figure 4).

**Figure 5:**
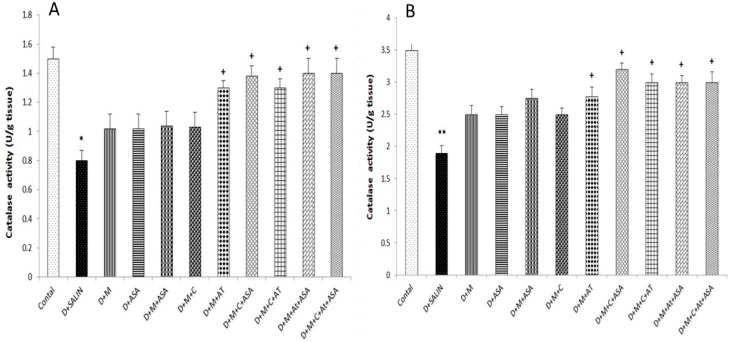
The catalase activity in the brain cortex (**A**) and hippocampus (**B**) tissues. Data were shown as mean ± SEM. * *p* < 0.05 and ** *p* < 0.01 compared to control and + *p* < 0.05 compared to diabetic group. D, diabetic; M, metformin; ASA, aspirin; AT, atorvastatin; and C, captopril.

Finally, the catalase activity in brain cortex (*p* < 0.05) and hippocampus (*p* < 0.01) in diabetic rats significantly decreased compared to the control group. All groups treated with captopril, aspirin, atorvastatin, metformin and their combinations potentiated catalase activity compared to the diabetic group, but this increase was significant only in (M + AT), (M + C + ASA), (M + C + AT), (M + AT + ASA) and (M + C + AT +ASA) groups (*p* < 0.05) (Figure 5).

## 4. Discussion

This study shows that the combined administration of all considered drugs produced more beneficial effects on glucose levels compared to the administration of individual drugs. In groups treated with metformin (D + M) or groups that received combinations of metformin and captopril (D + M + C), atorvastatin (D + M + AT) or aspirin (D + M + ASA), the total cholesterol, LDL-cholesterol and HDL-cholesterol levels significantly improved in contrast to the (D + ASA) group. This suggests that aspirin alone has no positive effect on lipid profile improvement. However, some recent studies reported that high-dose aspirin influences lipid metabolism [26,27].

In the present study, metformin improved the lipid profile when administered alone or in combination with captopril. Previous studies have demonstrated that metformin, as an anti-hyperglycaemic drug [28,29], and captopril can improve glucose and lipid metabolism [30]. In addition, the combination of metformin and atorvastatin (D + M + AT) reduced total cholesterol and LDL-cholesterol to a greater extent compared to the combination of metformin and aspirin (D + M + ASA) or captopril (D + M + C). Combination of metformin and atorvastatin (D + M + AT) significantly reduced TG in comparison with groups that received the combination of metformin and captopril and aspirin (D + M + C + ASA). Comparison of the groups that received combinations of three drugs demonstrated that in groups where atorvastatin was present (i.e., D + M + C + AT and D + M + ASA + AT groups), there was a significant decrease in TG levels compared to the treatment regimens that did not include atorvastatin (i.e., D + M + C + ASA group) suggesting high efficacy of atorvastatin in improving lipid profiles similar to previous studies [31].

Oxygen–glucose deprivation (OGD) in neurons increases extracellular glutamate levels leading to toxicity. Glutamate uptake from the synaptic space by glutamate transporters is altered by oxidative stress. Oxidative stress is associated with decreased activity of glutamate transporters as well as glutamine synthase, thereby increasing extracellular glutamate concentrations that may aggravate damage to neurons [32].

The possible mechanisms by which oxidative damage is involved in the pathophysiology of diabetes include activation of transcription factors, protein kinase C and advanced glycated end products (AGEs) [33]. Enhancement of nitric oxide (NO) generation in the brain in diabetes state induces nitrosative damage as well oxidative stress and the combination of NO with ROS resulting in the formation of a very toxic complex of peroxynitrite (ONOO-) that yields to the protein nitrotyrosination and cell death [34].

Studies have demonstrated the neuroprotective effect of angiotensin receptor blockers [35]. Mogi et al. demonstrated the preventive effects of telmisartan, a specific AT1 inhibitor, on cognitive and memory impairment in mice with Alzheimer disease induced by diabetes. Therefore, RAS inhibition by AT1 receptor antagonists or ACE inhibitors that are commonly used as anti-hypertensive drugs will be potentially able to prevent neurodegenerative diseases [36].

Abbasi et al. have been indicated that inhibition of RAS by using special inhibitors, valsartan (specific AT1 inhibitor) and captopril (ACE inhibitor), potentiates the antioxidant defense system of the brain. There was decreased oxidative/nitrosative stress as well as improvement of memory and cognitive function in neuronal damage during AD in diabetic rats. In this study, treatment with captopril as well as valsartan increased SOD and catalase activities [37]. This suggests that captopril has an antioxidant effect on the brain and hippocampus in diabetic rats. Therefore, the reduction of oxidative stress in the hippocampus can potentially lead to the improvement of memory and cognitive function. Enhancement in oxidative damage markers may be due to the increased Ang II formation and ACE activity, which stimulates NADPH oxidase, the key enzyme in the production of ROS, and plays a crucial role in the progression of oxidative stress [37].

Previous studies have been shown that the treatment of diabetes with metformin significantly increased the antioxidant enzymes’ activities and generally potentiated the antioxidant defense system in these cases [38,39]. Metformin prevents the oxidative stress consequences on apoptosis and inhibits the mitochondria-related toxicity of hyperglycaemia. In addition, the oxidative stress in endothelial cells was completely prevented by metformin. Treatment with metformin significantly increased glutathione levels. This finding reinforces the idea that the antidiabetic agent, metformin, has an important antioxidant function in the nervous system [40]. Therefore, this study demonstrated the antioxidant effects of metformin on the brain as well as its anti-hyperglycemic effects.

Aspirin, an NSAID with neuroprotective effects, inhibits brain iNOS expression as well as oxidative damage and ATP loss induced by immobilisation stress [41]. Castilli et al. have shown that low doses of aspirin has direct neuroprotective effects in patients with cerebral ischemia [42]. Also Moro et al. have demonstrated that ASA has potential neuroprotective effects by various mechanisms including inhibition of NF-kB translocation to the nucleus, interference with the mechanism leading to IkB phosphorylation and inhibition of oxidative stress [43]. Oxidative stress may activate the cytoplasmic NF-kB leading to its translocation to the nucleus [44]. Furthermore, inhibition of NF-kB activation by ASA has neuroprotective actions against neurotoxicity induced by glutamate [45].

Atorvastatin, a cholesterol-lowering drug, also has neuroprotective effects. Neuroprotective effects of statins such as atorvastatin may confer significant clinical benefit [46,47]. Studies have suggested that the putative anti-inflammatory and antioxidant properties of statins may confer additional neuroprotection in patients with cerebral ischaemia [48]. As a result, atorvastatin-induced neuroprotection may be associated with the reduction of oxidative stress. It inhibited OGD production as well as ROS. The addition of cholesterol before OGD and reoxygenation abolished the neuroprotective effect of atorvastatin as well as on glutamine synthetase and glutamate uptake activity. Furthermore, atorvastatin is capable of preventing cell death induced by OGD via amelioration of glutamine synthetase and glutamate uptake activity and reduces oxidative stress. Moreover, the effects of atorvastatin were related to its function on cholesterol synthesis inhibition. Indeed, atorvastatin could be a useful agent in the prevention of glutamate toxicity involved in brain damages such as vascular diseases [32]. The studies have been shown that increased oxidative stress in the parietal lobe of the cerebral cortex was related to poorer learning. Therefore, novel pharmacological effects of atorvastatin mediated by decreasing oxidative stress may be an important mechanism underlying the benefits of this agent. Generally, statins such as atorvastatin with higher blood–brain barrier penetrance have antioxidant effects and beneficial effects on cognition [49]. There are several neuroprotective pathways that could be affected by statins including BDNF production and activation of the PKB/Akt, Wnt and ERK pathways. Increased BDNF levels may activate PI3-kinase, which in turn activates PKB/Akt. PKB/Akt phosphorylates GSK-3β, thus reducing inhibition of the Wnt-signalling pathway. Also, statins can activate PKB/Akt by inhibiting PTEN (through Rho and Rho kinase) [50,51,52].

Our results demonstrated a significant increase of catalase and SOD activity in all groups and total thiol concentration in most groups that received different combinations of aspirin, atorvastatin, metformin and captopril. These results are in line with some previous studies that showed a combination of drugs exhibits additive effects on the reduction of oxidative stress. For example, Koh et al. and Qin et al. illustrated that a combination of statins with the inhibitors of the angiotensin II system exhibited a more potent reducing effect on oxidative stress [53,54]. Also, Giorgia et al. investigated putative additive effects of aspirin and statins in diabetes. Their study provided important information regarding the preventive role of aspirin in diabetes when used with statins to control cardiovascular risk factors [55]. Our findings showed that the combination of aspirin with atorvastatin, metformin and captopril, which are commonly consumed by patients with diabetes, potentiated the antioxidant effects of these medications and reduced oxidative stress. Further investigations are warranted to investigate the impact of emerging drug classes [56,57,58] and their combination with conventionala gents on oxidative stress and other aspects of neurological function in diabetic patients as well as other patient groups at a high risk of cardiovascular disease.

## 5. Conclusions

This study showed that combined use of atorvastatin, metformin, captopril and aspirin in diabetic rats potentiate their antioxidant effects on the brain and maybe have a beneficial effect on the cognitive functions possibly by neuroprotective effects on hippocampus area.

## References

[1] Ruggenenti P., Porrini E.L., Gaspari F., Motterlini N., Cannata A., Carrara F., Cella C., Ferrari S., Stucchi N., Parvanova A. (2012). Glomerular hyperfiltration and renal disease progression in type 2 diabetes. Diabetes Care.

[2] Wulsin L.R., Horn P.S., Perry J.L., Massaro J.M., D’agostino R.B. (2015). Autonomic imbalance as a predictor of metabolic risks, cardiovascular disease, diabetes, and mortality. J. Clin. Endocrinol. Metab..

[3] Lipinski B. (2001). Pathophysiology of oxidative stress in diabetes mellitus. J. Diabetes Its Complicat..

[4] Petrie J.R., Chaturvedi N., Ford I., Brouwers M.C., Greenlaw N., Tillin T., Hramiak I., Hughes A.D., Jenkins A.J., Klein B.E. (2017). Cardiovascular and metabolic effects of metformin in patients with type 1 diabetes (REMOVAL): A double-blind, randomised, placebo-controlled trial. Lancet Diabetes Endocrinol..

[5] Mousavi S.M., Niazmand S., Hosseini M., Hassanzadeh Z., Sadeghnia H.R., Vafaee F., Keshavarzi Z. (2015). Beneficial effects of Teucrium polium and metformin on diabetes-induced memory impairments and brain tissue oxidative damage in rats. Int. J. Alzheimers Dis..

[6] Wu L., Parhofer K.G. (2014). Diabetic dyslipidemia. Metabol.-Clin. Exp..

[7] Chehade J.M., Gladysz M., Mooradian A.D. (2013). Dyslipidemia in type 2 diabetes: Prevalence, pathophysiology, and management. Drugs.

[8] Chruściel P., Sahebkar A., Rembek-Wieliczko M., Serban M.C., Ursoniu S., Mikhailidis D.P., Jones S.R., Mosteoru S., Blaha M.J., Martin S.S. (2016). Impact of statin therapy on plasma adiponectin concentrations: A systematic review and meta-analysis of 43 randomized controlled trial arms. Atherosclerosis.

[9] Sahebkar A., Kotani K., Serban C., Ursoniu S., Mikhailidis D.P., Jones S.R., Ray K.K., Blaha M.J., Rysz J., Toth P.P. (2015). Statin therapy reduces plasma endothelin-1 concentrations: A meta-analysis of 15 randomized controlled trials. Atherosclerosis.

[10] Sahebkar A., Serban C., Mikhailidis D.P., Undas A., Lip G.Y., Muntner P., Bittner V., Ray K.K., Watts G.F., Hovingh G.K. (2015). Association between statin use and plasma d-dimer levels: A systematic review and meta-analysis of randomised controlled trials. Thromb. Haemost..

[11] Sahebkar A., Serban C., Ursoniu S., Mikhailidis D.P., Undas A., Lip G.Y., Bittner V., Ray K.K., Watts G.F., Hovingh G.K. (2016). The impact of statin therapy on plasma levels of von Willebrand factor antigen: Systematic review and meta-analysis of Randomised placebo-controlled trials. Thromb. Haemost..

[12] Serban C., Sahebkar A., Ursoniu S., Mikhailidis D.P., Rizzo M., Lip G.Y., Hovingh G.K., Kastelein J.J., Kalinowski L., Rysz J. (2015). A systematic review and meta-analysis of the effect of statins on plasma asymmetric dimethylarginine concentrations. Sci. Rep..

[13] Inoguchi T., Sonta T., Tsubouchi H., Etoh T., Kakimoto M., Sonoda N., Sato N., Sekiguchi N., Kobayashi K., Sumimoto H. (2003). Protein kinase C–dependent increase in reactive oxygen species (ROS) production in vascular tissues of diabetes: Role of vascular NAD (P) H oxidase. J. Am. Soc. Nephrol..

[14] Wassmann S., Laufs U., Bäumer A.T., Müller K., Ahlbory K., Linz W., Itter G., Rösen R., Böhm M., Nickenig G. (2001). HMG-CoA reductase inhibitors improve endothelial dysfunction in normocholesterolemic hypertension via reduced production of reactive oxygen species. Hypertension.

[15] Parizadeh S.M., Azarpazhooh M.R., Moohebati M., Nematy M., Ghayour-Mobarhan M., Tavallaie S., Rahsepar A.A., Amini M., Sahebkar A., Mohammadi M. (2011). Simvastatin therapy reduces prooxidant-antioxidant balance: Results of a placebo-controlled cross-over trial. Lipids.

[16] Calvin A.D., Aggarwal N.R., Murad M.H., Shi Q., Elamin M.B., Geske J.B., Fernandez-Balsells M.M., Albuquerque F.N., Lampropulos J.F., Erwin P.J. (2009). Aspirin for the primary prevention of cardiovascular events: A systematic review and meta-analysis comparing patients with and without diabetes. Diabetes Care.

[17] Ou H.-C., Lee W.-J., Wu C.-M., Chen J.F.-M., Sheu W.H.-H. (2012). Aspirin prevents resistin-induced endothelial dysfunction by modulating AMPK, ROS, and Akt/eNOS signaling. J. Vasc. Surg..

[18] Bolterman R.J., Manriquez M.C., Ruiz M.C.O., Juncos L.A., Romero J.C. (2005). Effects of captopril on the renin angiotensin system, oxidative stress, and endothelin in normal and hypertensive rats. Hypertension.

[19] Hundal R.S., Petersen K.F., Mayerson A.B., Randhawa P.S., Inzucchi S., Shoelson S.E., Shulman G.I. (2002). Mechanism by which high-dose aspirin improves glucose metabolism in type 2 diabetes. J. Clin. Investig..

[20] Heeba G.H., Hassan M.K., Amin R.S. (2009). Gastroprotective effect of simvastatin against indomethacin-induced gastric ulcer in rats: Role of nitric oxide and prostaglandins. Eur. J. Pharmacol..

[21] Abbasnezhad A., Niazmand S., Mahmoudabady M., Soukhtanloo M., Rezaee S.A., Mousavi S.M. (2016). Nigella sativa seed decreases endothelial dysfunction in streptozotocin-induced diabetic rat aorta. Avicenna J. Phytomed..

[22] Sedlak J., Lindsay R.H. (1968). Estimation of total, protein-bound, and nonprotein sulfhydryl groups in tissue with Ellman’s reagent. Anal. Biochem..

[23] Mihara M., Uchiyama M. (1978). Determination of malonaldehyde precursor in tissues by thiobarbituric acid test. Anal. Biochem..

[24] Madesh M., Balasubramanian K. (1998). Microtiter plate assay for superoxide dismutase using MTT reduction by superoxide. Indian J. Biochem. Biophys..

[25] Zini A., Lamirande E., Gagnon C. (1993). Reactive oxygen species in semen of infertile patients: Levels of superoxide dismutase-and catalase-like activities in seminal plasma and spermatozoa. Int. J. Androl..

[26] Dervisevik M., Dinevska-Kovkarovska S., Dimitrovska M., Cipanovska N., Miova B. (2014). High dose of aspirin moderates diabetes-induced changes of heart glycogen/glucose metabolism in rats. J. Physiol. Sci..

[27] Fullerton M.D., Ford R.J., McGregor C.P., LeBlond N.D., Snider S.A., Stypa S.A., Day E.A., Lhoták Š., Schertzer J.D., Austin R.C. (2015). Salicylate improves macrophage cholesterol homeostasis via activation of Ampk. J. Lipid Res..

[28] Wulffelé E.M., Kooy A., De Zeeuw D., Stehouwer C., Gansevoort R. (2004). The effect of metformin on blood pressure, plasma cholesterol and triglycerides in type 2 diabetes mellitus: A systematic review. J. Intern. Med..

[29] Zhou G., Myers R., Li Y., Chen Y., Shen X., Fenyk-Melody J., Wu M., Ventre J., Doebber T., Fujii N. (2001). Role of AMP-activated protein kinase in mechanism of metformin action. J. Clin. Investig..

[30] Malmqvist K., Kahan T., Isaksson H., Östergren J. (2001). Regression of left ventricular mass with captopril and metoprolol, and the effects on glucose and lipid metabolism. Blood Press..

[31] Kawamoto S., Kawamura T., Miyazaki Y., Hosoya T. (2007). Effects of atorvastatin on hyperlipidemia in kidney disease patients. Nihon Jinzo Gakkai Shi.

[32] Vandresen-Filho S., Martins W.C., Bertoldo D.B., Mancini G., Herculano B.A., Andreza F., Tasca C.I. (2013). Atorvastatin prevents cell damage via modulation of oxidative stress, glutamate uptake and glutamine synthetase activity in hippocampal slices subjected to oxygen/glucose deprivation. Neurochem. Int..

[33] Maritim A.C., Sanders A., Watkins J.B. (2003). Diabetes, oxidative stress, and antioxidants: A review. J. Biochem. Mol. Toxicol..

[34] Cetin F., Yazihan N., Dincer S., Akbulut G. (2013). The effect of intracerebroventricular injection of beta amyloid peptide (1–42) on caspase-3 activity, lipid peroxidation, nitric oxide and NOS expression in young adult and aged rat brain. Turk Neurosurg..

[35] Thöne-Reineke C., Zimmermann M., Neumann C., Krikov M., Li J., Gerova N., Unger T. (2004). Are angiotensin receptor blockers neuroprotective?. Curr. Hypertens. Rep..

[36] Mogi M., Li J.M., Tsukuda K., Iwanami J., Min L.J., Sakata A., Fujita T., Iwai M., Horiuchi M. (2008). Telmisartan prevented cognitive decline partly due to PPAR-γ activation. Biochem. Biophys. Res. Commun..

[37] Abbassi Y.A., Mohammadi M.T., Foroshani M.S., Sarshoori J.R. (2016). Captopril and valsartan may improve cognitive function through potentiation of the brain antioxidant defense system and attenuation of oxidative/nitrosative damage in STZ-induced dementia in rat. Adv. Pharm. Bull..

[38] Alhaider A.A., Korashy H.M., Sayed-Ahmed M.M., Mobark M., Kfoury H., Mansour M.A. (2011). Metformin attenuates streptozotocin-induced diabetic nephropathy in rats through modulation of oxidative stress genes expression. Chem.-Biol. Interact..

[39] Cahova M., Palenickova E., Dankova H., Sticova E., Burian M., Drahota Z., Cervinkova Z., Kucera O., Gladkova C., Stopka P. (2015). Metformin prevents ischemia reperfusion-induced oxidative stress in the fatty liver by attenuation of reactive oxygen species formation. Am. J. Physiol.-Gastrointest. Liver Physiol..

[40] Correia S., Carvalho C., Santos M.S., Proenca T., Nunes E., Duarte A.I., Monteiro P., Seica R., Oliveira C.R., Moreira P.I. (2008). Metformin protects the brain against the oxidative imbalance promoted by type 2 diabetes. Med. Chem..

[41] De Cristóbal J., Madrigal J.L.M., Lizasoain I., Lorenzo P., Leza J.C., Moro M.A. (2002). Aspirin inhibits stress-induced increase in plasma glutamate, brain oxidative damage and ATP fall in rats. Neuroreport.

[42] Castillo J., Leira R., Moro M.Á., Lizasoain I., Serena J., Dávalos A. (2003). Neuroprotective effects of aspirin in patients with acute cerebral infarction. Neurosci. Lett..

[43] Kopp E., Ghosh S. (1994). Inhibition of NF-kappa B by sodium salicylate and aspirin. Science.

[44] Schreck R., Meier B., Männel D.N., Dröge W., Baeuerle P.A. (1992). Dithiocarbamates as potent inhibitors of nuclear factor kappa B activation in intact cells. J. Exp. Med..

[45] Grilli M., Pizzi M., Memo M., Spano P. (1996). Neuroprotection by aspirin and sodium salicylate through blockade of NF-κB activation. Science.

[46] Bösel J., Gandor F., Harms C., Synowitz M., Harms U., Djoufack P.C., Megow D., Dirnagl U., Hörtnagl H., Fink K.B. (2005). Neuroprotective effects of atorvastatin against glutamate-induced excitotoxicity in primary cortical neurones. J. Neurochem..

[47] Lee S.-H., Kim Y.-H., Kim Y.-J., Yoon B.-W. (2008). Atorvastatin enhances hypothermia-induced neuroprotection after stroke. J. Neurol. Sci..

[48] Vaughan C.J., Delanty N., Basson C.T. (2001). Do statins afford neuroprotection in patients with cerebral ischaemia and stroke?. Cns Drugs.

[49] Barone E., Cenini G., Di Domenico F., Martin S., Sultana R., Mancuso C., Murphy M.P., Head E., Butterfield D.A. (2011). Long-term high-dose atorvastatin decreases brain oxidative and nitrosative stress in a preclinical model of Alzheimer disease: A novel mechanism of action. Pharmacol. Res..

[50] Van der Most P.J., Dolga A.M., Nijholt I.M., Luiten P.G., Eisel U.L. (2009). Statins: Mechanisms of neuroprotection. Prog. Neurobiol..

[51] Moro M.A., De Alba J., Cárdenas A., De Cristóbal J., Leza J.C., Lizasoain I., Dıaz-Guerra M.J., Boscá L., Lorenzo P. (2000). Mechanisms of the neuroprotective effect of aspirin after oxygen and glucose deprivation in rat forebrain slices. Neuropharmacology.

[52] El-Mir M.Y., Detaille D., Gloria R., Delgado-Esteban M., Guigas B., Attia S., Fontaine E., Almeida A., Leverve X. (2008). Neuroprotective role of antidiabetic drug metformin against apoptotic cell death in primary cortical neurons. J. Mol. Neurosci..

[53] Koh K.K., Son J.W., Ahn J.Y., Kim D.S., Jin D.K., Kim H.S., Han S.H., Seo Y.H., Chung W.J., Kang W.C. (2004). Simvastatin combined with ramipril treatment in hypercholesterolemic patients. Hypertension.

[54] Qin J., Zhang Z., Liu J., Sun L., Hu L., Cooper M.E., Cao Z. (2003). Effects of the combination of an angiotensin II antagonist with an HMG-CoA reductase inhibitor in experimental diabetes. Kidney Int..

[55] De Berardis G., Sacco M., Evangelista V., Filippi A., Giorda C.B., Tognoni G., Valentini U., Nicolucci A. (2007). Aspirin and Simvastatin Combination for Cardiovascular Events Prevention Trial in Diabetes (ACCEPT-D): Design of a randomized study of the efficacy of low-dose aspirin in the prevention of cardiovascular events in subjects with diabetes mellitus treated with statins. Trials.

[56] Yaribeygi H., Butler A.E., Barreto G.E., Sahebkar A. (2019). Antioxidative potential of antidiabetic agents: A possible protective mechanism against vascular complications in diabetic patients. J. Cell. Physiol..

[57] Sahebkar A., Watts G.F. (2013). New therapies targeting apoB metabolism for high-risk patients with inherited dyslipidaemias: what can the clinician expect?. Cardiovasc. Drugs Ther..

[58] McFadyen J.D., Peter K. (2017). Novel Antithrombotic Drugs on the Horizon: The Ultimate Promise to Prevent Clotting While Avoiding Bleeding. Circ. Res..

